# Commentary: Chilean gender identity support program in children and adolescents: review of a public health policy

**DOI:** 10.3389/fpubh.2026.1935897

**Published:** 2026-09-22

**Authors:** Gabriel Bastías, Cristian Borgoño, M. Alejandra Carrasco, Juan Pablo Rojas, Mónica Silva, Francisca Ugarte

**Affiliations:** 1 Escuela de Salud Pública, Pontificia Universidad Católica de Chile, Santiago, Chile; 2 Facultad de Teología, Pontificia Universidad Católica de Chile, Santiago, Chile; 3 Instituto de Filosofía, Universidad de los Andes, Santiago, Chile; 4 Escuela de Psicología, Universidad Finis Terrae, Santiago, Chile; 5 Academia Senior UC, Pontificia Universidad Católica de Chile, Santiago, Chile; 6 Facultad de Medicina, Universidad de los Andes, Santiago, Chile; 7 Clínica Universidad de los Andes, Santiago, Chile

**Keywords:** gender identity, gender identity policies, gender identity programs, gender non-conforming children and adolescents, gender-affirmative programs, parental rights

## Introduction

A recent paper by Martinez et al.—“Chilean Gender Identity Support Program [PAIG] in Children and Adolescents: Review of a Public Health Policy” (1)—describes a public initiative “explicitly designed to support trans and gender non-conforming children, adolescents and their families.”

This commentary highlights three unaddressed or misrepresented issues in the article. First, PAIG is framed within a gender-affirmative model, rather than a neutral exploratory support program, and its age range—from 3 to 17 years—raises developmental issues. Second, parental engagement in the program is valued, but is contingent on parental approval of the gender-affirmative interventions offered. Third, although PAIG is formally described as a psychosocial intervention, some minors admitted to PAIG received hormonal interventions with public funding.

We address program shortcomings and suggest a rigorous evaluation to ensure it meets the quality and ethical standards of a national evidence-based public health policy.

## A gender-affirming program

PAIG (2) is an intersectoral program that involves the Ministry of Health, the Ministry of Social Development and the Ministry of Education. Chile's territorial healthcare system is organized into 29 Health Services and provides an appropriate framework for the implementation and delivery of health programs, such as PAIG, through an integrated care network that enables planned access and timely referrals.

PAIG is based on a gender-affirmative framework (3–5) and seeks “to provide guidance to children and adolescents affirming their gender identity… provide orientation for caretakers… to attain acceptance and support in the affirmation of their child's [chosen] gender identity…” (2).^1^ Thus, affirmation is the organizing principle of PAIG's interventions.

PAIG was approved in 2021 (2) and implemented in 2023 (6, 7), a time when mounting evidence brought into question the scientific foundations of gender-affirmative interventions in minors (8–11). Such evidence was overlooked in PAIG's design. Conversely, during that period several countries reassessed and adjusted their clinical guidelines (12–14). Subsequently, the Cass Review (15) concluded that the evidence base for the gender-affirming model was deficient and prompted a departure from this approach in the UK toward a broader model of care, with attention to mental health, family context, and long-term follow-up.

As a program, PAIG covers a broad age range from 3 to 17 years (2), spanning markedly different physical, cognitive, emotional, and social developmental stages (16, 17). However, PAIG does not provide specific approaches tailored to the developmental characteristics of each age group. For example, differentiated intervention strategies, communication methods, and decision-making processes, should have been designed for children aged 3–9 years, 10–13 years, and 14–17 years.^2^ This consideration is particularly important for interventions such as social transition, where developmental needs and outcomes may vary substantially across age groups.

Most gender-nonconforming (GNC) prepuberal children do not persist as such by adolescence or adulthood (18–23). Early social transition is an intervention that assumes that gender identity is fixed at an early age and disregards the key role of neuroplasticity in childhood and adolescent development (24). Thus, early social transitioning may alter a child's trajectory by increasing persistence rates (18, 24, 25). Since persistence may entail a lifelong dependency on medication and surgeries, early social transitioning may not be innocuous.

Considering the child's best interest and the lack of robust evidence on the risks and benefits of early social transitions (25–27), the program should reconsider this practice to prevent future psychological consequences associated with detransition (27–30).

Delaying social transitions could prevent non-persisting GNC youth from having to make a complex change back to their birth role and gender, as the proponents of the early Dutch protocol acknowledged (30, 31) and the main clinical international guideline recommends (32).

## Parental legal role

The role of parents entails a set of affective, social and legal responsibilities aimed at nurturing their child's comprehensive development (33). As in other nations, parental rights are legally protected in Chile's Constitution (34), which guarantees their preferential right and duty to educate, care, protect and guide their children (35). Also, Chilean Law 20.584 (36) regulates patients' rights and duties, establishing that parents or legal guardians must give informed consent for health interventions performed on minor children.^3^

Since its inception, PAIG has been marked by an unresolved tension between two principles: a child's right to be heard and to exercise progressive autonomy, and the parents' legal authority and responsibility to make decisions on behalf of their children, including decisions related to education and healthcare. Consistent with the child's right to be heard, the Ministry of Education issued two normative decrees requiring educational institutions to address children and adolescents by their preferred social name, regardless of parental disapproval (37, 38). Parental authority was further limited until December 2025, as any child or adolescent could request admission to the PAIG program without parental consent. Currently, minors under the age of 14 require informed consent from at least one parent or legal guardian to participate in the program (7).

Although Martinez et al. underscore the “fundamental role of families”—and PAIG offers psychosocial family support and orientation—in practice the parental role is challenged. PAIG's “Technical Orientations” document states that if parents or tutors oppose the child's or adolescent's expressed wishes, or if there is a suspicion of rights infringement, the team within the public health system “can undertake the appropriate management and/or legal actions,”^4^ actions that can eventually compromise child custody (7). The downside to this leeway to pursue legal actions is that parental disagreement with PAIG interventions can be misconstrued as neglect, maltreatment, or rights infringement.

The program's prioritization of children's rights and decisions about gender identity interventions over parental rights has been highly debated and renders it susceptible to legitimate criticism and legal actions.^5^

## Hormone delivery

Martinez et al. state that PAIG does not provide hormone interventions and merely consists of psychosocial support (1). However, a journalistic investigation revealed that puberty blockers and hormone affirmative medications were prescribed to GNC youngsters in public and private medical centers (39). In response to the public outcry and commotion that followed its publication, the Chamber of Deputies^6^ appointed an investigative commission (CIE 57) to scrutinize PAIG protocols, their application, and the program's administration (40). In this context, the Minister of Health acknowledged that approximately 600 minors enrolled in PAIG received hormonal treatment: 68% were 15–18 years old and 32% were 10–14 years old. Despite the weak evidence of its benefits and unknown long term adverse effects (15, 40), treatments were funded by the Health Ministry, through connected or parallel pathways (40).

The Final Report of CIE 57, approved by the Chamber of Deputies on November 2024, recommended a halt to new treatments to protect GNC minors. Other recommendations included a review of demographic and clinical data, a revision of parental consents, a reformulation of the guidelines to prevent hormonal therapies for adolescents under 18 years, and the provision of specialized care to those who seek to detransition (40).

## PAIG as a public health program

As Martinez et al. acknowledge, one of PAIG's main weaknesses is the limited availability of clinical outcomes and follow-up data (1). However, the problem is more profound: The program was implemented without baseline diagnostic information, and no prevalence data on GNC Chilean children and adolescents were available at the time (40), thereby limiting reliable estimates of population needs and program coverage. Furthermore, PAIG lacks clearly defined diagnostic criteria for admission and operates without standardized instruments and protocols to detect risk factors, assess mental health, appraise participant capacity for informed consent, assess the quality of family functioning, and evaluate participants' social and educational environment (2, 7).

Owing to the work conducted by CEI 57, some limited descriptive PAIG information became available, such as number of entrants and recipients of hormonal treatment. The program, after 3 years of implementation, still lacks a functional central monitoring system to characterize program entrants and track their outcomes over time. Patient data tracking should include baseline demographic characteristics, reasons for referral, psychological and psychiatric assessment, baseline gender-related stress, comorbidities, referrals to other medical services, family and parental functioning and participation, specific interventions provided, school context information, adverse events, changes or reversals in social transition, medical treatment (where applicable), waiting time for admission, continuity of care, and discharge criteria, among others. Finally, longitudinal data is required on adverse events, discontinuation, and detransition-related needs. Without it, assessing program effectiveness or impact is not possible.

PAIG's shortcomings in its evaluative framework cannot be attributed to a national shortage of resources nor to an inadequate local evaluative capacity. In fact, other intersectoral programs—e.g. “*Habilidades para la Vida*” (“Life Skills”)—provide a useful local reference for comparison. “Life Skills”^7^ is a public, intersectoral psychosocial and mental health initiative. Since its inception, it has included standardized screening, repeated measurements, and longitudinal evaluation of educational outcomes, demonstrating that evaluative capacity is available and can be embedded within a Chilean intersectoral public program (41, 42).

Additionally, an analysis of the PAIG program reveals a paradigmatic case of a policy implementation gap (43), i.e., a substantial discrepancy between its formal and explicit design and what occurs in practice. PAIG may be formally described as a rights-based and psychosocial program (2), while real-world implementation involves heterogeneous gender-affirming practices (6, 7, 31, 44), with unclear referral pathways, uneven safeguards, and limited accountability. In this regard, as with any health program, PAIG should be evaluated by what it does, not merely by what it declares to do.

## Final remarks

We agree with Martinez et al. on the necessity to have a national program to address the needs of GNC children and adolescents, and their families (1, 45). However, PAIG as a public health program has important limitations, as also reported by others (46). To attribute criticism of the program to political and religious motivations, as Martinez et al. do (1), is misleading and undermines the need for change: PAIG's core principles were laid out under a center-right government led by a Catholic president. Moreover, PAIG's restrictions of hormonal interventions in minors were approved at de Chamber of Deputies with votes from politicians representing the full political spectrum (right, center, and left) (47).

Finally, a rigorous external evaluation of PAIG remains pending. Controversial programs are particularly vulnerable to polarization, politicization, conflicts of interest, and efforts to control information (48). PAIG requires a complete redesign and a critical data-based evaluation of its practices. There is a pressing need to establish evidence-based guidelines, as done in Finland (49) and elsewhere. The aim in Chile should be to develop a program that complies with the high standards expected of any national public health policy.

## Footnotes

1 Authors' translation. Original text in Spanish reads “El objetivo es entregar orientación al NNA afirmando su identidad de género… entregar orientación a los cuidadores … de tal manera de lograr su aceptación y apoyo a la afirmación de la identidad género de su hijo(a)” (pp. 5–6).

2 In Chile legal adulthood is reached at 18 years of age.

3 Chile's Political Constitution (Art. 19 N° 10), Law 21.430 “Guarantees and Protection of Children and Adolescents Rights” (Title II, Art. 10), Law 20.584 “Informed Consent in Healthcare” (Title II, Paragraph 7°, Art.14).

4 Authors' translation. Original text in Spanish reads “o se sospeche de alguna situación de vulneración de derechos, el equipo deberá realizar las acciones de gestión clínica, administrativa y/o legales que correspondan” (p. 22).

5 A local non-governmental organization (NGO) –*Comunidad y Justicia*–has provided legal advice in 6 cases and represented 5 parents who were summoned or sued in the local courts (Lawyer Javier Mena, Personal Communication 17/6/2026).

6 The Chamber of Deputies operates as the lower house of Chile's national legislature, akin to the “House of Representatives” in other bicameral systems like the U.S.A. and Australia.

7 The program is run by the National Board of School Aid and Scholarships (JUNAEB). It originated from joint research with the Ministry of Health Mental Unit and operates in conjunction with the health sector for specialized care.
